# A new family of azanaphthoquinones for antimicrobial evaluation

**DOI:** 10.1186/s13065-018-0388-3

**Published:** 2018-02-23

**Authors:** Nilüfer Bayrak

**Affiliations:** Chemistry Department, Engineering Faculty, Istanbul University, 34320 Avcilar, Istanbul Turkey

**Keywords:** Azanaphthoquinone, Aromatic amines, Antimicrobial activity

## Abstract

This article presents a complete and detailed study of synthesis, structural characterization, and possible applications of a new family of azanaphthoquinones as antimicrobial agents. A series of (alkoxy)phenylamino-chloro-2-methylquinoline-5,8-dione derivatives (**3a**–**j**, **3a′**, **3e′**) was prepared by regioselective nucleophilic substitution of 6,7-dichloro-2-methylquinoline-5,8-dione (**1**) with (alkoxy)arylamines (**2**) in the presence of CeCl_3_·7H_2_O. In vitro antimicrobial study of the newly synthesized compounds was evaluated in a panel of three fungi and seven bacterial strains (three Gram-positive and four Gram-negative bacteria). As a result, the compounds (**3a**, **3b**, and **3h**) were identified as the hits with the strong antibacterial efficiency against the human originated pathogens *S. epidermidis* and *E. faecalis* with some minimal inhibitory concentration values. The antibacterial activity of the compound (**3h**) was two times more active against *S. epidermidis* than the reference antimicrobial compound (Cefuroxime). Two compounds (**3a** and **3b**) exhibited excellent antibacterial activity (four times more active than Cefuroxime) against *S. epidermidis*. In addition to *S. epidermidis*, these three compounds (**3a**, **3b**, and **3h**) were more active against *E. faecalis* than the reference antimicrobial compound (Amikacin). The antibacterial activity of the compounds (**3a** and **3h**) was three times more active against *E. faecalis*. The compound (**3b**) was long dozen times more active against *E. faecalis*. For that reason, these three compounds (**3a**, **3b**, and **3h**) were thought to be considered as the promising antibacterial agents.

## Introduction

Looking at the outline of the existing literature on my research area to find out what are the reasons for the great importance of the compounds retaining quinoline-5,8-dione (QD) skeleton, one main section is clearly discernible. Structural and biological aspects of these derivatives make them attractive for highly successful applications, especially in pharmaceutical fields [1, 2]. These compounds are valuable and functional structures due to their possible biological activities including antibacterial, antifungal, anticancer, antimalarial, and antiviral activities [3–8]. QD skeleton has been introduced as a ‘privileged template’ to be a consequence of fragment-based drug discovery approaches (FDDA) that have already been penetrated into both pharmaceutical industry and academia on a large scale as an alternative methodology instead of combinatorial chemistry and high-throughput screening (HTS) techniques [9]. According to FDDA, some structural subunits that commonly found in bioactive compounds or natural substances are especially useful in design of bioeffectors [10, 11]. Evaluated by such a perspective of prevailing conceptual view, QD fulfills the requirement not only because of the existence of the skeleton in lavendamycin, and streptonigrin, a natural product family called “streptonigrinoids” but also due to their antimicrobial activity [12, 13]. The synthetic routes towards the important precursors of QD and its congeners have become involved in organic chemistry with special focus on a nascent class of antimicrobials.

Antimicrobials are used as a weapon to fight infectious diseases caused by germs having four main kinds, bacteria, viruses, fungi, and protozoa [14, 15]. Since these living organisms could be everywhere like air, soil, and water, the infections could easily spread by touching, eating, or even breathing [15]. According to the list of top 10 causes of death in the world that is declared by World Health Organization (WHO), infectious diseases such as lower respiratory tract infections, HIV/AIDS, and tuberculosis especially effecting the people most particularly in low- and middle-income countries were at the top in 2015 [16]. The principal reason for this situation is antimicrobial resistance which is described as the alteration of microorganisms at the time of their subjection to antimicrobial drugs and is usually created by the mis- and/or overuse of antimicrobials [17]. In February 2017, WHO reported a list of antibiotic-resistant priority pathogens as the greatest threat to human health containing Gram-positive and negative bacteria like *Pseudomonas aeruginosa*, *Enterococcus faecium*, *Staphylococcus aureus*, *Klebsiella pneumonia*, *Escherichia coli* in order to address new antibiotics that are emergently required and to encourage researchers to develop new antibiotics [17].

Our previous work on targeting synthesizing disubstituted-1,4-naphthoquinones containing a substituted arylamine and evaluating in vitro antimicrobial potential of them against Gram-positive and negative bacteria strains and fungi has led to the results that some compounds were identified as the hits with the strong antibacterial efficiency against the human originated pathogens *S. epidermidis* exhibiting excellent antibacterial activity [18]. When the toxicity of some compounds was compared with the reference clinically proven antimicrobial drug Cefuroxime, the antibacterial activities of some compounds were 2–4 times more active than Cefuroxime [18]. By the evaluation of the studies related to the structure–activity relationships on QD and its derivatives, the clear evidence was observed that the antimicrobial activity has been associated with the structural diversity at the benzyl chain or/and at the quinoline ring of the QD core. Based on the facts infections are among the top ten causes of morbidity and mortality worldwide and due to the high degree of resistance of germs by the way putting a bridle on the therapy of the infections, I have paid particular attention to synthesize new organic compounds comprising QD skeleton as antimicrobial drug candidates with the hope of circumventing the vital threats and creating new antibiotics that are urgently needed.

## Results and discussion

### Chemistry

Keeping in mind the previous researches on the regioselective nucleophilic substitution of dichloro quinoline-5,8-dione with amines, new 6-arylamino-7-chloro-5,8-quinazolinedione analogues have been synthesized using CeCl_3_·7H_2_O and ethanol as reported before [19]. What we do know from existing literature is that the nucleophilic substitution of dichloro-2-methylquinoline-5,8-dione compound (**1**) containing two unsymmetrically substituted chlorine atoms which are capable of replacing amines can occur at the C-6 and/or C-7 position depending on the reaction parameters. By adding a Lewis acid like CeCl_3_·7H_2_O, the regioselectivity of 6-position is significantly increased owing to decreased electron density at C-6 via the chelation of Ce(III) between heterocyclic nitrogen atom and oxygen atom at C-8 position. As a result, the chlorine atom in position 6 is more inclinable to be substituted than that in position 7 [19]. Besides, polar solvents such as ethanol are also known to enhance the formation of 6-isomer products.

**Scheme 1:**
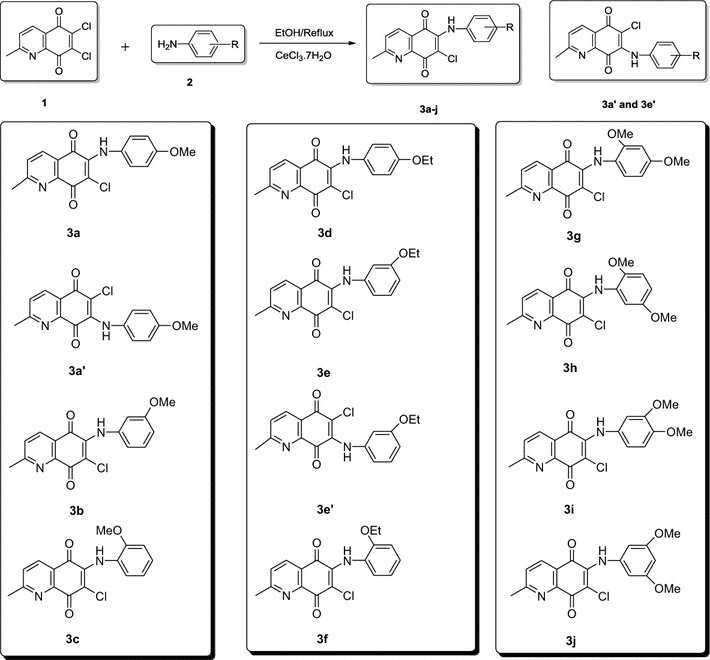
The library of new family of azanaphthoquinones

Starting material 6,7-dichloro-2-methylquinoline-5,8-dione (**1**) was synthesized according to a procedure described in the literature [20]. The quinoline **1** was reacted analogously with commercial amines such as 4-methoxybenzenamine, 3-methoxybenzenamine, 2-methoxybenzenamine, 4-ethoxybenzenamine, 3-ethoxybenzenamine, 2-ethoxybenzenamine, 2,4-dimethoxybenzenamine, 2,5-dimethoxybenzenamine, 3,4-dimethoxybenzenamine, and 3,5-dimethoxybenzenamine to give 6-alkoxyphenylamino-7-chloroquinoline diones (**3a**–**j**) as the main products except two compounds named **3a′** and **3e′** that are 7-alkoxyphenylamino-6-chloroquinoline diones. The reaction mixtures of products (**3a**–**j**, **3a′**, and **3e′**) were then subjected for purification using column chromatography. The chemical structures of the derivatives were confirmed by ^1^H and ^13^C NMR, IR, and MS methods. The synthesis of compounds (**3a**–**j**, **3a′**, and **3e′**) were depicted in Scheme 1.

As a matter of fact, it is observed the characteristic signals of the IR spectra of **3a**–**j**, **3a′**, **3e′** showed characteristic absorptions at around 3200 cm^−1^ (NH); 1650 cm^−1^ (C=O), 2900 cm^−1^ (CH_aliphatic_). The ^1^H NMR spectra of all synthesized compounds exhibited the NH signals as a singlet at around 7.60 ppm. The aromatic proton resonances appeared in a range of 6–8 ppm. The signals related to other functional groups observed in the expected regions.

### Antimicrobial activity

A previous study was aimed to investigate the interaction of 6-chloroquinoline-5,8-dione hydrochloride and 6,7-dichloroquinoline-5,8-quinone with sulfa drugs such as sulfadiazine, sulfadimidine, and sulfathiazole. In that study, the effect of products characterized as 7-substituted sulfonamide 10H-pyrido[3,2-b]carbazole-5,11-dione and 6-substituted sulfonamide derivatives of 7-chloroquinoline-5,8-quinone on some Gram positive and Gram negative bacterial species were tested [21]. To better understand how the antifungal activity might be influenced by both the number and the position of halogen or other substituents on the aniline ring, it would be helpful to look over the reports related to the synthesis of 6-[*N*-(substitutedphenyl)amino]-7-chloro-5,8-quinolinediones to use for their antifungal susceptibility testings, in vitro, against pathogenic *Candida* species and bacteria. Some derivatives of these compounds showed more potent antifungal activities than ketoconazole, fluconazole and griseofulvin used for antifungal references in these studies. Most of derivatives were found to be more active than ampicillin against gram-positive bacteria. Moreover, 6-[(*N*-2,3-dichlorophenyl)amino]-7-chloro-5,8-quinolinedione was tested for their in vivo antifungal activities in the treatment of systemic infection with *Candida albicans* in normal mice compared with ketoconazole and it was declared as a potent antifungal agent [4, 22].

To explore the antibacterial potential of the synthesized QDs, total twelve compounds were evaluated in vitro for their antibacterial activity against gram-positive and gram-negative bacteria and for their antifungal activity against fungi using the microbroth dilution technique according to the Clinical Laboratory Standards Institute (CLSI) recommendations [23, 24]. The antimicrobial assay results of all the newly synthesized QDs (**3a**–**j**, **3a′**, and **3e′**) are given in Table 1. Concerning the antibacterial activity, the results showed that some compounds displayed versatile effects on the growth of the tested gram-positive and gram-negative bacterial strains. The test-cultures *P. aeruginosa* and *P. mirabilis* appeared not to be susceptible to synthesized compounds. Findings shown in Table 1 revealed that compounds have exhibited moderate activity against both Gram-positive bacteria. All of the synthesized compounds possessed activity against *S. aureus* and *S. epidermidis* with MIC values of between 2.44 and 1250 μg/mL. Additionally, all of the compounds, except **3a′**, and **3e′**, possessed activity against *E. faecalis* with MIC values of between 9.76 and 625 μg/mL. Compounds **3a**, **3b**, and **3h** showed excellent activity against *S. epidermidis* and *E. faecalis*. These results are important considering the intrinsic resistance to Cefuroxime and Amikacin of *S. epidermidis* and *E. faecalis*, respectively. These three compounds showed fairly wide antifungal spectra. The activities of the compounds **3a**, **3b**, and **3h** were superior as compared to that of Cefuroxime. The compounds **3a**, **3b**, and **3h**, completely inhibited the growth of *S. epidermidis*, was tested at the MIC level of 2.44, 2.44, and 4.88 μg/mL, respectively. By contrast, 7-isomer products of **3a** and **3e**, namely **3a′** and **3e′**, has no significant activity against *S. epidermidis* and *E. faecalis*. In addition to these, the activities of compounds  **3g** and **3j** showed moderate activity against *S. epidermidis*.

**Table 1:** In vitro antibacterial and antifungal activity results of the synthesized QDs

		Microorganisms									
		Gram-negative bacteria				Gram-positive bacteria			Fungi		
		P. aeruginosa	E. coli	K. pneumoniae	P. mirabilis	S. aureus	S. epidermidis	E. faecalis	C. albicans	C. parapsilosis	C. tropicalis
MIC values (μg/mL)	**3a**	–	156.2	–	–	156.2	*2.44*	*39.06*	78.12	78.12	39.06
	**3a′**	–	–	–	–	312.5	156.2	–	312.5	–	–
	**3b**	–	312.5	–	–	78.12	*2.44*	*9.76*	39.06	9.76	156.2
	**3c**	–	–	–	–	39.06	39.06	156.2	–	–	312.5
	**3d**	–	–	–	–	78.12	625	625	–	–	–
	**3e**	–	156.2	–	–	312.5	39.06	312.5	–	–	312.5
	**3e′**	–	–	–	–	312.5	312.5	–	–	–	312.5
	**3f**	–	312.5	–	–	312.5	1250	625	–	78.12	–
	**3g**	–	156.2	–	–	312.5	19.53	625	–	156.2	–
	**3h**	–	–	625	–	39.06	*4.88*	*39.06*	–	156.2	312.5
	**3i**	–	–	–	–	312.5	312.5	625	–	78.12	312.5
	**3j**	–	312.5	625	–	39.06	19.53	312.5	–	156.2	312.5
	Reference Antimicrobials	2.4 Ceftazidime	4.9 Cefuroxime-Na	4.9 Cefuroxime-Na	2.4 Cefuroxime-Na	1.2 Cefuroxime-Na	9.8 Cefuroxime	128 Amikacin	4.9 Clotrimazole	0.5 Amphotericin B	1 Amphotericin B

The lowest values are in italics

To evaluate the importance of the position and alkyl chain of alkoxy group (–OR) on the phenyl amine ring with respect to the biological efficiency, the activities of all the synthesized compounds were compared (Scheme 1). It was found that replacing the methoxy group (–OCH_3_) position in the synthesized compounds (**3a**–**c**) from the meta position to the para position or the meta position to the ortho position led to activity loss. Additionally, replacing the methoxy group (–OCH_3_) by an ethoxy group (–OCH_2_CH_3_) in all positions did not show any positive progress on activity. Presence of the two methoxy groups in different positions such as 2,4-, 2,5-, 3,4-, and 3,5-(**3g**–**j**) led to a decrease in activity when compared with compounds (**3a**–**c**) containing only one methoxy group. On the other hand, presence of the two methoxy groups (**3g**–**j**) has still much more active than the compounds (**3d**–**f**) containing ethoxy group. According to this data, we can conclude that, in general, the QDs containing one methoxy group showed better antibacterial activity than their ethoxy derivatives.

## Materials and methods

### Chemistry

All reagents and solvents were commercially purchased from different companies and used as supplied. Chemical reactions were monitored by using aluminium-based plates (silica gel 60 F254) from Merck. Plates were visualized under UV light (254 nm). Column chromatographic separations were carried out using silica gel 60 (Merck, 63–200 μm particle size, 60–230 mesh). Melting points (mp) were determined with a Buchi B-540 melting point without being corrected. ^1^H NMR and ^13^C NMR spectra were recorded with either VarianUNITY INOVA spectrometers with 500 MHz frequency for ^1^H and 125 MHz frequency for ^13^C NMR or BRUKER spectrometers with 400 MHz frequency for ^1^H and 100 MHz frequency for ^13^C NMR in ppm. ^1^H NMR spectra and ^13^C NMR spectra in CDCl_3_ refer to the solvent signal center at 7.19 and 76.0 ppm, respectively. Chemical shifts are expressed in ppm downfield relative to tetramethylsilane. Data for ^1^H NMR spectra are reported as follows: s (singlet), br s (broad singlet), d (doublet), t (triplet), q (quartet), dd (doublet of doublets), and m (multiplet) and the coupling constants J are given in Hz. IR spectra were recorded as ATR on either Thermo Scientific Nicolet 6700 spectrometer or Alpha T FTIR spectrometer. Mass spectra were obtained on either a Thermo Finnigan LCQ Advantage MAX MS/MS spectrometer equipped with ESI (electrospray ionization) sources. (Alkoxyphenylamino)-chloro-2-methylquinoline-5,8-dione derivatives (**3a**–**j**, **3a′**, and **3e′**) were prepared from the reactions of 6,7-dichloro-2-methylquinoline-5,8-dione (**1**) with alkoxy substituted aryl amines (**2a**–**j**) according to the procedure previously reported [19].

#### General procedure for synthesis of the (alkoxyphenylamino)-chloro-2-methylquinoline-5,8-dione derivatives (**3a**–**j**, **3a′**, **3e′**)

Into a 100 mL round bottom flask were added 250 mg (1.00 mmol) of 6,7-dichloro-2-methylquinoline-5,8-dione (**1**), ethanol (30 mL), CeCl_3_·7H_2_O, and alkoxy substituted aryl amines (**2**), respectively. The reaction mixture was stirred at room temperature until thin layer chromatography (TLC) showed the absence of the starting materials. The resulting solution was extracted with 100 mL chloroform then washed with water (3 × 100 mL) and dried over calcium chloride. The solvent was removed *in vacuo*. The residue was subjected to column chromatography on silica gel using suitable solvents to give the products (**3a**–**j**, **3a′**, and **3e′**) [19].

##### 6-(4-Methoxyphenylamino)-7-chloro-2-methylquinoline-5,8-dione (**3a**)

It was synthesized from 6,7-dichloro-2-methylquinoline-5,8-dione (**1**) and 4-methoxybenzenamine (**2a**) as purple powder by using the general procedure. Yield: 0.1 g, 30%, mp: 189–190 °C. FTIR (ATR) ν(cm^−1^): 3234 (–NH), 2967, 2938, 2855 (CH_aliphatic_), 1678, 1649 (C=O), 1580 (C=C). ^1^H NMR (500 MHz, *CDCl*_*3*_) δ (ppm): 8.30 (d, *J*: *7.8* *Hz*, 1H, –CH_arom_), 7.63 (br s, 1H, –NH), 7.48 (d, *J*: *7.8* *Hz*, 1H, –CH_arom_), 7.10–7.04 (m, 2H, –CH_arom_), 6.94–6.86 (m, 2H, –CH_arom_), 3.85 (s, 3H, –OCH_3_), 2.80 (s, 3H, –CH_3_). ^13^C NMR (125 MHz, *CDCl*_*3*_) δ(ppm): 180.1, 176.1 (C=O), 166.0, 158.0, 148.1, 141.2, 134.9, 130.9, 126.7, 126.3, 124.6, 114.4, 113.7 (C_q_ and C_arom_), 55.5 (–OCH_3_), 25.4 (–CH_3_). MS (ESI+) *m/z* (%): 328 (100, [M]^+^). Anal. Calcd. for C_17_H_13_ClN_2_O_3_ (328.75).

Additionally, **3a′** was also obtained from 6,7-dichloro-2-methylquinoline-5,8-dione (**1**) and 4-methoxybenzenamine (**2a**) as purple powder by using the general procedure.

##### 7-(4-Methoxyphenylamino)-6-chloro-2-methylquinoline-5,8-dione (**3a′**)

Yield: 0.034 g, 10%. mp: 215–216 °C. FTIR (ATR) ν(cm^−1^): 3226 (–NH), 3002, 2969, 2851 (CH_aliphatic_), 1678, 1652 (C=O), 1609, 1580 (C=C). ^1^H NMR (400 MHz, *CDCl*_*3*_) δ (ppm): 8.31 (d, *J*: *8.0* *Hz*, 1H, –CH_arom_), 7.67 (br s, 1H, –NH), 7.48 (d, *J*: *8.0* *Hz*, 1H, –CH_arom_), 7.00 (d, *J*: *8.8* *Hz*, 2H, –CH_arom_), 6.81 (d, *J*: *8.9* *Hz*, 2H, –CH_arom_), 3.50 (s, 3H, –OCH_3_), 2.70 (s, 3H, –CH_3_). ^13^C NMR (100 MHz, *CDCl*_*3*_) δ(ppm): 179.4, 176.7 (C=O), 164.0, 158.0, 146.0, 142.0, 135.1, 130.0, 128.6, 128.0, 126.5, 113.6, 112.5 (C_q_ and C_arom_), 55.5 (–OCH_3_), 25.1 (–CH_3_). MS (ESI+) *m/z* (%): 329 (100, [M + H]^+^), 331 (32, [M + 3H]^+^). Anal. Calcd. for C_17_H_13_ClN_2_O_3_ (328.75).

##### 6-(3-Methoxyphenylamino)-7-chloro-2-methylquinoline-5,8-dione (**3b**)

It was synthesized from 6,7-dichloro-2-methylquinoline-5,8-dione (**1**) and 3-methoxybenzenamine (**2b**) as purple oil by using the general procedure. Yield: 0.050 g, 15%. FTIR (ATR) ν(cm^−1^): 3426 (–NH), 2971, 2934, 2985, 2859 (CH_aliphatic_), 1678, 1652, (C=O), 1576 (C=C). ^1^H NMR (400 MHz, *CDCl*_*3*_) δ (ppm): 8.34 (d, *J*: *8.0* *Hz*, 1H, –CH_arom_), 7.67 (br s, 1H, –NH), 7.52 (d, *J*: *8.0* *Hz*, 1H, –CH_arom_), 7.28 (t, *J*: *8.1 Hz*, 1H, –CH_arom_), 6.82 (dd, *J*: *8.2*, *J*: *2.3* *Hz*, 1H, –CH_arom_), 6.72 (dd, *J*: *9*,*8*, *J*: *2.0* *Hz*, 1H, –CH_arom_), 6.65 (t, *J*: *2.2* *Hz*, 1H, –CH_arom_), 3.84 (s, 3H, –OCH_3_), 2.82 (s, 3H, –CH_3_). ^13^C NMR (100 MHz, *CDCl*_*3*_) δ(ppm): 180.1, 176.2 (C=O), 166.1, 159.7, 148.0, 140.9, 138.3, 135.0, 129.2, 126.9, 124.7, 116.8, 116.3, 111.7, 110.2 (C_q_ and C_arom_), 55.5 (–OCH_3_), 25.5 (–CH_3_). MS (ESI+) *m/z* (%): 329 (100, [M + H]^+^), 331 (28, [M + 3H]^+^). Anal. Calcd. for C_17_H_13_ClN_2_O_3_ (328.75).

##### 6-(2-Methoxyphenylamino)-7-chloro-2-methylquinoline-5,8-dione (**3c**)

It was synthesized from 6,7-dichloro-2-methylquinoline-5,8-dione (**1**) and 2-methoxybenzenamine (**2c**) as dark red powder by using the general procedure. Yield: 0.179 g, 53%, mp: 210–211 °C. FTIR (ATR) ν(cm^−1^): 3224 (–NH), 2998, 2994, 2845 (CH_aliphatic_), 1676, 1652 (C=O), 1607, 1576 (C=C). ^1^H NMR (400 MHz, *CDCl*_*3*_) δ (ppm): 8.33 (d, *J*: *7.9* *Hz*, 1H, –CH_arom_), 7.63 (br s, 1H, –NH), 7.51 (d, *J*: *7.9* *Hz*, 1H, –CH_arom_), 7.27–7.22 (m, 1H, –CH_arom_), 7.07–6.92 (m, 3H, –CH_arom_), 3.88 (s, 3H, –OCH_3_), 2.82 (s, 3H, –CH_3_). ^13^C NMR (100 MHz, *CDCl*_*3*_) δ(ppm): 180.0, 176.1 (C=O), 166.0, 152.6, 148.1, 141.2, 135.0, 126.8, 126.1, 125.2, 124.8, 119.9, 115.4, 110.7 (C_q_ and C_arom_), 55.7 (–OCH_3_), 25.6 (–CH_3_). MS (ESI+) *m/z* (%): 329 (100, [M + H]^+^), 331 (22, [M + 3H]^+^). Anal. Calcd. for C_17_H_13_ClN_2_O_3_ (328.75).

##### 6-(4-Ethoxyphenylamino)-7-chloro-2-methylquinoline-5,8-dione (**3d**)

It was synthesized from 6,7-dichloro-2-methylquinoline-5,8-dione (**1**) and 4-ethoxybenzenamine (**2d**) as purple powder by using the general procedure. Yield: 0.165 g, 47%, mp: 193–194 °C. FTIR (ATR) ν(cm^−1^): 3224 (–NH), 2998, 2969, 2855 (CH_aliphatic_), 1676, 1652 (C=O), 1607, 1578 (C=C). ^1^H NMR (400 MHz, *CDCl*_*3*_) δ (ppm): 8.21 (d, *J*: *8.00* *Hz*, 1H, –CH_arom_), 7.52 (br s, 1H, –NH), 7.40 (d, *J*: *8.0* *Hz*, 1H, –CH_arom_), 7.16–7.07 (m, 1H, –CH_arom_), 7.00–6.92 (m, 1H, –CH_arom_), 6.88–6.78 (m, 2H, –CH_arom_), 4.00 (q, *J*: *13.9*, *6.9* *Hz*, 2H, –OCH_2_), 2.71 (s, 3H, –CH_3_), 1.27 (t, *J*: *7.0* *Hz*, 3H, –CH_3_). ^13^C NMR (100 MHz, *CDCl*_*3*_) δ(ppm): 179.9, 176.0 (C=O), 165.9, 152.0, 148.1, 141.4, 134.9, 126.7, 126.3, 125.1, 124.8, 119.8, 115.5, 111.6 (C_q_ and C_arom_), 64.2 (–OCH_2_), 25.5, 14.8 (–CH_3_). MS (ESI−) *m/z* (%): 341 (100, [M − H]^−^), 343 (30, [M + H]^−^). Anal. Calcd. for C_18_H_15_ClN_2_O_3_ (342.78).

##### 6-(3-Ethoxyphenylamino)-7-chloro-2-methylquinoline-5,8-dione (**3e**)

It was synthesized from 6,7-dichloro-2-methylquinoline-5,8-dione (**1**) and 3-ethoxybenzenamine (**2e**) as dark red powder by using the general procedure. Yield: 0.157 g, 44%, mp: 187–188 °C. FTIR (ATR) ν(cm^−1^): 3330 (–NH), 2969, 2928, 2861 (CH_aliphatic_), 1678, 1666 (C=O), 1603, 1580 (C=C). ^1^H NMR (500 MHz, *CDCl*_*3*_) δ (ppm): 8.22 (d, *J*: *7.8* *Hz*, 1H, –CH_arom_), 7.55 (br s, 1H, –NH), 7.40 (d, *J*: *7.8* *Hz*, 1H, –CH_arom_), 7.16 (t, *J*: *8.3* *Hz*, 1H, –CH_arom_), 6.69 (dd, *J*: *8.3*, *J*: *2.4* *Hz*, 1H, –CH_arom_), 6.59 (dd, *J*: *7.8*, *J*: *1.9* *Hz*, 1H, –CH_arom_), 6.54–6.53 (m, 1H, –CH_arom_), 3.98–3.93 (m, 2H, –OCH_2_), 2.71 (s, 3H, –CH_3_), 1.33 (t, *J*: *6.8* *Hz*, 3H, –CH_3_). ^13^C NMR (125 MHz, *CDCl*_*3*_) δ(ppm): 179.1, 175.1 (C=O), 165.1, 158.1, 147.0, 139.9, 137.3, 133.9, 128.1, 125.8, 123.7, 115.7, 115.0, 111.3, 109.8 (C_q_ and C_arom_), 62.7 (–OCH_2_), 24.4, 13.8 (–CH_3_). MS (ESI−) *m/z* (%): 341 (100, [M − H]^−^), 343 (32, [M + H]^−^). Anal. Calcd. for C_18_H_15_ClN_2_O_3_ (342.78).

Additionally, **3e′** was also obtained from 6,7-dichloro-2-methylquinoline-5,8-dione (1) and 3-ethoxybenzenamine (**2e**) as red oil by using the general procedure.

##### 7-(3-Ethoxyphenylamino)-6-chloro-2-methylquinoline-5,8-dione (**3e′**)

Yield: 0.010 g, 3%. FTIR (ATR) ν(cm^−1^): 3442 (−NH), 2965, 2926, 2857, (CH_aliphatic_), 1637 (C=O), 1509, 1466 (C=C). ^1^H NMR (500 MHz, *CDCl*_*3*_) δ (ppm): 8.31 (d, *J*: *8.3* *Hz*, 1H, –CH_arom_), 7.69 (br s, 1H, –NH), 7.47 (d, *J*: *7.8* *Hz*, 1H, –CH_arom_), 7.17 (t, *J*: *7.8* *Hz*, 1H, –CH_arom_), 6.70 (dd, *J*: *8.3*, *2.4* *Hz*, 1H, –CH_arom_), 6.62 (dd, *J*: *7.8*, *1.9* *Hz*, 1H, –CH_arom_), 6.56–6.55 (m, 1H, –CH_arom_), 3.98–3.91 (m, 2H, –OCH_2_), 2.70 (s, 3H, –CH_3_), 1.34 (t, *J*: *6.8* *Hz*, 3H, –CH_3_). ^13^C NMR (125 MHz, *CDCl*_*3*_) δ(ppm): 178.3, 175.7 (C=O), 163.1, 158.1, 144.7, 140.8, 137.2, 134.1, 128.1, 127.4, 115.9, 111.4, 109.9, 106.8, 103.7 (C_q_ and C_arom_), 62.7 (–OCH_2_), 24.0, 13.8 (–CH_3_). MS (ESI+) *m/z* (%): 343 (100, [M + H]^+^), 345 (33, [M + 3H]^+^). Anal. Calcd. for C_18_H_15_ClN_2_O_3_ (342.78).

##### 6-(2-Ethoxyphenylamino)-7-chloro-2-methylquinoline-5,8-dione (**3f**)

It was synthesized from 6,7-dichloro-2-methylquinoline-5,8-dione (1) and 2-ethoxybenzenamine (**2f**) as purple powder by using the general procedure. Yield: 0.20 g, 56%, mp: 205–206 °C. FTIR (ATR) ν(cm^−1^): 3334 (–NH), 3002, 2947, 2908 (CH_aliphatic_), 1672, 1607 (C=O), 1580 (C=C). ^1^H NMR (400 MHz, *CDCl*_*3*_) δ (ppm): 8.33 (d, *J*: *8.0* *Hz*, 1H, –CH_arom_), 7.66 (br s, 1H, –NH), 7.51 (d, *J*: *8.0* *Hz*, 1H, –CH_arom_), 7.26–7.17 (m, 1H, –CH_arom_), 7.08–7.02 (m, 1H, –CH_arom_), 6.91–7.01 (m, 2H, –CH_arom_), 4.11 (q, *J*: *14.0*, *7.0* *Hz*, 2H, –OCH_2_), 2.83 (s, 3H, –OCH_3_), 1.39 (t, *J*: *7.0* *Hz*, 3H, –CH_3_). ^13^C NMR (100 MHz, *CDCl*_*3*_) δ(ppm): 180.0, 176.1 (C=O), 165.9, 152.0, 148.1, 141.3, 134.9, 126.7, 126.3, 125.1, 124.9, 119.8, 115.6, 111.6 (C_q_ and C_arom_), 64.2 (–OCH_2_–), 25.5, 14.8 (–CH_3_). MS (ESI−) *m/z* (%): 341 (100, [M − H]^−^), 343 (22, [M + H]^−^). Anal. Calcd. for C_18_H_15_ClN_2_O_3_ (342.78).

##### 6-(2,4-Dimethoxyphenylamino)-7-chloro-2-methylquinoline-5,8-dione (**3g**)

It was synthesized from 6,7-dichloro-2-methylquinoline-5,8-dione (**1**) and 2,4-dimethoxybenzenamine (**2g**) as brown powder by using the general procedure. Yield: 0.074 g, 20%, mp: 186–187 °C. FTIR (ATR) ν(cm^−1^): 3224 (–NH), 3000, 2996, 2865 (CH_aliphatic_), 1676, 1652 (C=O), 1607, 1580 (C=C). ^1^H NMR (400 MHz, *CDCl*_*3*_) δ (ppm): 8.31 (d, *J*: *7.8* *Hz*, 1H, –CH_arom_), 7.49 (m, 2H, –CH_arom_ and –NH), 7.02 (d, *J*: *9.4* *Hz*, 1H, –CH_arom_), 6.53–6.48 (m, 2H, –CH_arom_), 3.88 (s, 3H, –OCH_3_), 3.83 (s, 3H, –OCH_3_), 2.78 (s, 3H, CH_3_). ^13^C NMR (100 MHz, *CDCl*_*3*_) δ(ppm): 180.0, 176.0 (C=O), 165.9, 159.3, 154.4, 148.2, 141.6, 134.9, 126.7, 126.6, 124.8, 119.4, 114.2, 103.4, 98.7 (C_q_ and C_arom_), 55.7 (–OCH_3_), 25.5 (–CH_3_). MS (ESI+) *m/z* (%): 359 (100, [M + H]^+^), 361 (34, [M + 3H]^+^). Anal. Calcd. for C_18_H_15_ClN_2_O_4_ (358.78).

##### 6-(2,5-Dimethoxyphenylamino)-7-chloro-2-methylquinoline-5,8-dione (**3h**)

It was synthesized from 6,7-dichloro-2-methylquinoline-5,8-dione (**1**) and 2,5-dimethoxybenzenamine (**2h**) as purple powder by using the general procedure. Yield: 0.248 g, 67%, mp: 176–177 °C. FTIR (ATR) ν(cm^−1^): 3226 (–NH), 2998, 2967, 2867 (CH_aliphatic_), 1676, 1652, (C=O), 1607, 1578. (C=C). ^1^H NMR (500 MHz, *CDCl*_*3*_) δ (ppm): 8.21 (d, *J*: *7.8* *Hz*, 1H, –CH_arom_), 7.49 (br s, 1H, –NH), 7.40 (d, *J*: *7.8* *Hz*, 1H, –CH_arom_), 6.75 (d, *J*: *8.8* *Hz*, 1H, –CH_arom_), 6.66 (dd, *J*: *8.8*, *J*: *2.9* *Hz*, 1H, –CH_arom_), 6.51 (d, *J*: *2.9* *Hz*, 1H, –CH_3_), 3.88 (s, 3H, –OCH_3_), 3.73 (s, 3H, –OCH_3_), 2.71 (s, 3H, CH_3_). ^13^C NMR (125 MHz, *CDCl*_*3*_) δ(ppm): 179.8, 176.0 (C=O), 165.9, 152.9, 148.3, 146.9, 141.1, 134.9, 134.8, 126.7, 126.6, 124.8, 115.8, 111.4, 111.3 (C_q_ and C_arom_), 55.8, 56.2 (–OCH_3_), 25.5 (–CH_3_). MS (ESI−) *m/z* (%): 357 (100, [M − H]^−^), 359 (22, [M + H]^−^). Anal. Calcd. for C_18_H_15_ClN_2_O_4_ (358.78).

##### 6-(3,4-Dimethoxyphenylamino)-7-chloro-2-methylquinoline-5,8-dione (**3i**)

It was synthesized from 6,7-dichloro-2-methylquinoline-5,8-dione (1) and 3,4-dimethoxybenzenamine (**2i**) as dark red oil by using the general procedure. Yield: 0.274 g, 74%. FTIR (ATR) ν(cm^−1^): 3224 (–NH), 3002, 2989, 2859 (CH_aliphatic_), 1676, 1652 (C=O), 1607, 1578 (C=C). ^1^H NMR (500 MHz, *CDCl*_*3*_) δ (ppm): 8.22 (d, *J*: *7.8* *Hz*, 1H, –CH_arom_), 7.57 (br s, 1H, –NH), 7.40 (d, *J*: *8.3* *Hz*, 1H, –CH_arom_), 6.76 (d, *J*: *8.8* *Hz*, 1H, –CH_arom_), 6.63–6.59 (m, 2H, –CH_arom_), 3.84 (s, 3H, –OCH_3_), 3.80 (s, 3H, –OCH_3_), 2.71 (s, 3H, CH_3_). ^13^C NMR (125 MHz, *CDCl*_*3*_) δ(ppm): 180.1, 176.1 (C=O), 166.1, 148.6, 148.1, 147.6, 141.1, 134.9, 130.2, 126.7, 124.6, 117.3, 114.6, 110.5, 109.2 (C_q_ and C_arom_), 56.1, 56.0 (–OCH_3_), 25.4 (–CH_3_). MS (ESI−) *m/z* (%): 357 (100, [M − H]^−^), 359 (38, [M + H]^−^). Anal. Calcd. for C_18_H_15_ClN_2_O_4_ (358.78).

##### 6-(3,5-Dimethoxyphenylamino)-7-chloro-2-methylquinoline-5,8-dione (**3j**)

It was synthesized from 6,7-dichloro-2-methylquinoline-5,8-dione (**1**) and 3,5-dimethoxybenzenamine (**2**) as dark red powder by using the general procedure. Yield: 0.190 g, 51%, mp: 197–198 °C. FTIR (ATR) ν(cm^−1^): 3226 (–NH), 3002, 2994, 2853 (CH_aliphatic_), 1678, 1652 (C=O), 1607, 1580 (C=C). ^1^H NMR (500 MHz, *CDCl*_*3*_) δ (ppm): 8.22 (d, *J*: *8.3* *Hz*, 1H, –CH_arom_), 7.52 (br s, 1H, –NH), 7.40 (d, *J*: *8.3* *Hz*, 1H, –CH_arom_), 6.26 (t, *J*: *1.9* *Hz*, 1H, –CH_arom_), 6.16 (d, *J*: *2.4* *Hz*, 2H, –CH_arom_), 3.71 (s, 6H, –OCH_3_), 2.71 (s, 3H, CH_3_). ^13^C NMR (125 MHz, *CDCl*_*3*_) δ(ppm): 180.0, 176.2 (C=O), 166.1, 160.5, 147.9, 140.9, 138.8, 134.9, 126.9, 124.7, 116.3, 103.00, 102.9, 98.2, 98.1 (C_q_ and C_arom_), 55.4, 55.6 (–OCH_3_), 25.5 (–CH_3_). MS (ESI+) *m/z* (%): 359 (100, [M + H]^+^). Anal. Calcd. for C_18_H_15_ClN_2_O_4_ (358.78).

### In vitro antimicrobial activity

#### Determination of minimum inhibitory concentrations (MIC)

Antimicrobial activity against *Staphylococcus aureus* ATCC 29213, *Staphylococcus epidermidis* ATCC 12228, *Escherichia coli* ATCC 25922, *Klebsiella pneumoniae* ATCC 4352, *Pseudomonas aeruginosa* ATCC 27853, *Proteus mirabilis* ATCC 14153, *Enterococcus faecalis* ATCC 29212, *Candida albicans* ATCC 10231, *Candida parapsilosis* ATCC 22019, and *Candida tropicalis* ATCC 750 was determined by the microbroth dilutions technique using the Clinical Laboratory Standards Institute (CLSI) recommendations [23, 24]. Mueller–Hinton broth for bacteria and RPMI-1640 medium for yeast strain were used as the test medium. Serial twofold dilutions ranging from 5000 to 2.44 µg/mL were prepared in medium. The inoculum was prepared using a 4–6 h broth culture of each bacteria type and 24 h culture of yeast strains adjusted to a turbidity equivalent to 0.5 McFarland standard, diluted in broth media to give a final concentration of 5 × 10^5^ cfu/mL for bacteria and 5 × 10^3^ cfu/mL for yeast in the test tray. The trays were covered and placed into plastic bags to prevent evaporation. The trays containing Mueller–Hinton broth were incubated at 35 °C for 18–20 h while the trays containing RPMI-1640 medium were incubated at 35 °C for 46–50 h. The MIC was defined as the lowest concentration of compound giving complete inhibition of visible growth. As control, antimicrobial effects of the solvents were investigated against test microorganisms. According to values of the controls, the results were evaluated. The MIC values of the compounds are given in Table 1.

## Conclusions

In summary, in order to obtain antimicrobial lead compounds, (alkoxy)phenylamino-chloro-2-methylquinoline-5,8-dione derivatives (**3a**–**j**, **3a′**, **3e′**) were synthesized. Three active compounds (**3a**, **3b**, and **3h**) exhibited better activity than that of the reference antimicrobial compound. In vitro antimicrobial study of the newly synthesized compounds was evaluated in a panel of three fungi and seven bacterial strains (three Gram-positive and four Gram-negative bacteria). As a result, the compounds (**3a**, **3b**, and **3h**) were identified as the hits with the strong antibacterial efficiency against the human originated pathogens *S. epidermidis* and *E. faecalis* with some minimal inhibitory concentration values. These results suggest that these substances have potential for exploring the design of new antimicrobial prototypes against Gram-positive bacteria. Moreover, the results might encourage the synthesis of new 6-substituted-5,8-quinolinedione analogs for improving potential. Comparing the activity of the methoxy and ethoxy derivatives of QD compounds, one can notice that the introduction of the amines with methoxy group leads to positive changes in the antibacterial activity against tested pathogens.
